# Blood HDAC4 Variation Links With Disease Activity and Response to Tumor Necrosis Factor Inhibitor and Regulates CD4+ T Cell Differentiation in Ankylosing Spondylitis

**DOI:** 10.3389/fmed.2022.875341

**Published:** 2022-05-06

**Authors:** Bin Dou, Fuzhe Ma, Zhenyu Jiang, Ling Zhao

**Affiliations:** ^1^Department of Rheumatology, The First Hospital of Jilin University, Changchun, China; ^2^Department of Nephrology, The First Hospital of Jilin University, Changchun, China

**Keywords:** Histone deacetylase 4, ankylosing spondylitis, disease activity, treatment outcome, T-helper 17 polarization

## Abstract

**Purpose:**

Histone deacetylase 4 (HDAC4) regulates the progression of autoimmune diseases. This study aimed to further investigate the correlation between HDAC4 and Th cells, inflammation, disease activity, and treatment response in patients with ankylosing spondylitis (AS).

**Methods:**

A total of 132 active patients with AS were enrolled, of whom 54 patients received TNF inhibitor (TNFi) and 78 patients received NSAID. Serum HDAC4 was measured by ELISA in patients with AS before treatment (W0) and at week (W)4, W8, and W12 after treatment. Meanwhile, serum HDAC4 was detected in 30 patients with osteoarthritis and in 30 healthy controls (HCs) by ELISA. Besides, naïve CD4^+^ T cells from patients with AS were isolated, followed by modulation of HDAC4 and then polarization toward Th1, Th2, and Th17.

**Results:**

Histone deacetylase 4 was reduced in patients with AS compared with HCs and patients with osteoarthritis (both *P* < 0.01). In patients with AS, HDAC4 was negatively correlated with TNF (*P* < 0.001), IL-1β (*P* = 0.003), Th17 proportion (*P* = 0.008), C-reactive protein (*P* < 0.001), and ASDAS (*P* = 0.038), but not with IL-6, Th1 proportion, or other characteristics. Meanwhile, HDAC4 increased from W0 to W12 (*P* < 0.001); HDAC4 at W8 (*P* = 0.014) and W12 (*P* = 0.006) was raised in ASAS40-response patients than ASAS40-non-response patients; further subgroup analysis showed that HDAC4 at W12 was higher in ASAS40-response patients than ASAS40-non-response patients (*P* = 0.016) in the TNFi-treated group, but not in the NSAID-treated group. In addition, HDAC4 negatively regulated the polarization of naïve CD4^+^ T cells toward Th17 (*P* < 0.01), but not Th1 or Th2.

**Conclusion:**

Histone deacetylase 4 is associated with lower inflammation, and the disease activity negatively regulates Th17 polarization, whose increment after treatment reflects favorable outcomes in patients with AS.

## Introduction

Ankylosing spondylitis (AS) is an autoimmune disease that more frequently affects men and is closely associated with the positivity of the human leukocyte antigen-B27 (HLA-B27) gene (1). It is estimated that around five million people in Asia are affected by AS; these individuals face not only back pain and decreased mobility of the spinal area but also high medical costs, reduced quality of life, and even inability to work (2–4). Currently, the primary treatment modality of AS is a non-steroidal anti-inflammatory drug (NSAID) and biologics [with tumor necrosis factor inhibitor (TNFi) as the primary type], alone or in combination, which shows valuable efficacy and improves the quality of life in patients with AS (5–7). However, some patients with AS may likely fail to respond to the treatments or face a high recurrence risk (8). Therefore, it is vital to pursue potential biological sites that could reflect disease conditions and, to some extent, indicate treatment response, which may possess a specific value to improve the long-term management of AS.

Histone deacetylase 4 (HDAC4), an enzyme that reduces the acetylation level of histones, is involved in modifying various gene expressions and exerts diverse biological functions; it has been reported as a critical regulator of complex diseases, such as cancers, stroke, and autoimmune diseases (9–12). Regarding the role of HDAC4 in autoimmune diseases, it was revealed that inhibition of HDAC4 promotes the proliferation and migration of fibroblast-like synoviocytes; it also exacerbates pathological damage in rheumatoid arthritis rats (12). Meanwhile, HDAC4 modulates inflammation in diverse pathological conditions, such as rheumatoid arthritis, asthma, and atherosclerosis, while inflammation is one of the hallmarks of autoimmune diseases (8, 13–15). Furthermore, HDAC4 can regulate the differentiation of T helper (Th) 17, which plays a vital role in the progression of AS by increasing the level of proinflammatory cytokines and thus directly aggravating AS (16–18); meanwhile, it is also suggested that Th17 participates in the bone remodeling in AS (19). However, apart from the experimental findings, few studies have explored the clinical implication of HDAC4 in autoimmune diseases such as AS, let alone its relevance to treatment response in AS.

This study aimed to evaluate the association of HDAC4 with inflammation, disease activity, treatment response, and its effect on Th cell polarization in patients with AS.

## Methods

### Participants

Between May 2019 and July 2021, this study serially included 132 active patients with AS. The inclusion criteria were as follows: (i) confirmed as AS according to Assessment of Spondylarthritis International Society (ASAS) criteria (20); (ii) age ≥18 years; (iii) Bath Ankylosing Spondylitis Disease Activity Index (BASDAI) score ≥4 [on a 0–10 cm Visual Analog Scale (VAS)]; (iv) Ankylosing Spondylitis Disease Activity Score (ASDAS) ≥2.1; (v) were about to receive TNFi treatment or NSAID treatment; and (vi) willing to comply with the study protocol for data collection, sample collection, and follow-up. The exclusion criteria were as follows: (i) concomitant with tuberculosis or hepatitis; (ii) had severe joint deformity; (iii) had a chronic or recurrent infection; (iv) complicated with cancer or hematologic malignancy; and (v) pregnant and lactating female patient.

In addition, during the same period, 30 patients with osteoarthritis (OA) were enrolled as disease controls (DCs) in the study. All patients with OA, aged over 18 years, were diagnosed by imageological examinations. Additionally, 30 healthy subjects were also recruited in the study as healthy controls (HCs). The DCs and HCs who had an infection, tuberculosis, hepatitis, severe joint deformity, cancer, or hematologic malignancy were excluded, and the subjects who were pregnant or lactating were also ineligible.

The written informed consents were collected from all subjects. The study was permitted by Ethics Committee.

### Collection of Data and Samples

For patients with AS, clinical characteristics were obtained after admission. Peripheral blood (PB) samples were collected from all 132 patients with AS at W0 (before treatment initiation), from 127 patients with AS at W4 (4 weeks after treatment initiation), from 116 patients with AS at W8 (8 weeks after treatment initiation), and from 104 patients with AS at W12 (12 weeks after treatment initiation). Then, peripheral blood mononuclear cell (PBMC) and serum samples were isolated. For DCs and HCs, PB samples were collected after recruitment, and then, PBMC samples were separated.

### Determination of Samples

The collected PBMC samples of all subjects were used to determine the expression of HDAC4 by reverse transcription-quantitative polymerase chain reaction (RT-qPCR). A total of 69 fresh PB samples of patients with AS were available to detect T-helper 1 (Th1) cell percentage and T-helper 17 (Th17) cell percentage by flow cytometry. Serum samples of all AS patients were used to assess the level of proinflammatory factors [e.g., tumor necrosis factor (TNF), interleukin (IL)-1β, and IL-6], the cytokine secreted by Th1 cells [interferon-gamma (IFN-γ)], and the cytokine secreted by Th17 cells (IL-17A) by enzyme-linked immunosorbent assay (ELISA).

### Treatment

A total of 54 patients with AS were treated with TNFi, including adalimumab and etanercept. The detailed regimens were as follows: adalimumab was administered subcutaneously at a dose of 40 mg every 2 weeks; etanercept was administered subcutaneously at a dose of 50 mg every week. Besides, a total of 78 patients with AS were treated with NSAID, including celecoxib, meloxicam, and imrecoxib. The detailed regimens were as follows: celecoxib was administered orally at a dose of 200 mg/day; meloxicam was administered orally at a dose of 7.5 mg/day; imrecoxib was administered orally at a dose of 100 mg two times a day.

### Evaluation

At W4, W8, and W12, treatment response was assessed according to the ASAS40 response criteria (20). During the follow-up, a total of 28 patients dropped out early from the study. Of these, 22 cases were lost to follow-up, three cases had poor efficacy, one case had an abnormal liver function, one case had an active infection, and one case was no longer in the study. For the assessment of the ASAS40 response, the missing data were processed using the last observation carried forward (LOCF) method. In the analysis, patients who achieved ASAS40 response at W12 were categorized as response patients, and the others without the achievement of ASAS40 response at W12 were categorized as non-response patients.

### Cell Isolation and Transfection

Human naïve CD4^+^ T cells were isolated from the PBMC of patients with AS using a naïve CD4^+^ T cell isolation kit (Miltenyi, Germany). Cells were maintained in GT-T551 T-cell culture and expansion medium (Takara, Japan) and transfected with HDAC4 overexpression lentivirus (HDAC4 group), HDAC4 knockdown lentivirus (anti-HDAC4 group), or control lentivirus (Scramble group) (Genepharma, China) according to the manual. Un-transfected naïve CD4^+^ T cells were used as the control (control group). After 48 h incubation, cells were harvested for detecting the expression of HDAC4 by RT-qPCR and Western blot.

### Cell Polarization

The polarization of Th1, T-helper 2 (Th2), and Th17 cells was performed as described previously (21, 22). Briefly, transfected naïve CD4^+^ T cells were stimulated with the following polarizing conditions for 3 days: IL-12 (10 ng/ml, Sigma, USA) and anti–IL-4 (5 μg/ml, Affinity, USA) for Th1 polarization; IL-4 (2.5 ng/ml, Sigma, USA) and anti-IFN-γ (5 μg/ml, Affinity, USA) for Th2 polarization; TGF-β (10 ng/ml, Sigma, USA), IL-6 (10 ng/ml, Sigma, USA), IL-1β (10 ng/ml, Sigma, USA), IL-23 (10 ng/ml, Sigma, USA), anti–IL-4 (5 μg/ml), and anti-IFN-γ (5 μg/ml) for Th17 polarization. After polarization, the cells and supernatants were collected for flow cytometry and ELISA, respectively.

### RT-qPCR

Total RNA of PBMC samples or transfected cells was purified using TRIzol (Invitrogen, USA) and quantified using NanoDrop (Thermo, USA). The cDNA Synthesis Kit (Bio-Rad, USA) and PCR Master Mix (Beyotime, China) were used for RT-qPCR according to the manufacturer's instructions. The expression of HADC4 was normalized to GAPDH using the 2^−ΔΔCt^ method. The primers used were as follows: HDAC4, 5'-AGAATGGCTTTGCTGTGGTC-3' (forward) and 5'-ATCTTGCTCACGCTCAACCT-3' (reverse); GAPDH, 5'-GAGTCCACTGGCGTCTTCAC-3' (forward) and 5'-ATCTTGAGGCTGTTGTCATACTTCT-3' (reverse).

### Western Blot

Total proteins were extracted using RIPA (Beyotime, China) and quantified with a protein quantification kit (Beyotime, China). The SDS-PAGE was performed for protein separation using 10% precast gels (Thermo, USA). The polyvinylidene difluoride membranes (Millipore, USA) were applied for protein transfer. After being blocked with 5% bovine serum albumin (BSA) buffer, the membrane was incubated with rabbit anti-HDAC4 antibody (1:2,000, Affinity, USA) or rabbit anti-GAPDH antibody (1:10,000, Affinity, USA). Bands were revealed using an ECL kit (Servicebio, China) after secondary antibody incubation (1:10,000, Affinity, USA). Quantification of HDAC4 was normalized to GAPDH by densitometry using ImageJ software (NIH, USA).

### Flow Cytometry

Th1 cell percentage and Th17 cell percentage among patients with AS were measured by flow cytometry using Human Th1/Th17 Phenotyping Kit (Thermo, USA), whereas in *in vitro* experiment, the percentages of Th1, Th2, and Th17 cells were determined using Human Th1/Th2/Th17 Phenotyping Kit (BD, USA). Briefly, PBMC samples or polarized cells were stimulated with ionomycin (5 μM, Sigma, USA), phorbol myristic acetate (PMA) (1 μM, Beyotime, China), and BD GolgiStop (1 μl/ml, BD, USA) for 6 h before collection. For PBMC samples, cells were then stained with FITC-conjugated anti-CD4, PE-conjugated anti-IFN-γ, and APC-conjugated anti-IL-17A. For polarized cells, cells were then stained with PerCP-Cy5.5-conjugated anti-CD4 and FITC-conjugated anti-IFN-γ for the assessment of Th1 cells; PerCP-Cy5.5-conjugated anti-CD4 and APC-conjugated anti-IL-4 for the assessment of Th2 cells; and PerCP-Cy5.5-conjugated anti-CD4 and APC-conjugated anti-IL-17A for the assessment of Th17 cells. After being stained for 0.5 h, cells were analyzed using a FACSCanto II (BD, USA). The data were analyzed using FlowJo7.6 software (BD, USA). Briefly, CD4^+^ cells were first gated, then IFN-γ^+^, IL-4^+^, and IL-17A^+^ cells were gated for calculating the percentages of Th1, Th2, and Th17 cells in CD4^+^ cells, respectively.

### ELISA

The levels of TNF, IL-1β, IL-6, IFN-γ, and IL-17A among patients with AS were determined by ELISA using a commercial Human ELISA Kit (Sangon Biotech, China). For *in vitro* experiment, ELISA detection was performed using Th1/Th2/Th17 cytokine Human ProcartaPlex™ Panel (Thermo, USA). The experimentations were performed according to the manuals provided by the manufacturers.

### Statistical Analysis

SPSS V.21.0 (IBM Corp., USA) was employed for data analysis. GraphPad Prism V.7.0 (GraphPad Software Inc., USA) was implemented for graph plotting. The difference of HDAC4 expression among the three groups was analyzed using the Kruskal-Wallis H rank sum test, followed by *post-hoc* comparisons using the Bonferroni test. The differentiation performance of HDAC4 expression was estimated using the receiver operating characteristic (ROC) curve. The correlation of HDAC4 expression with clinical characteristics was determined using Spearman's rank correlation test or Wilcoxon rank sum test. Change of HDAC4 expression over time was determined using the Friedman test. Comparison analysis between the two groups was executed using the chi-square test or Wilcoxon rank sum test. In the study of *in vitro* experiment, statistical significance among groups was evaluated using one-way ANOVA with Tukey's *post-hoc* test. *P*-value <0.05 was considered statistically significant.

## Results

### Clinical Characteristics

The mean age of the enrolled patients with AS was 37.1 ± 9.7 years with 14 (10.6%) female patients and 118 (89.4%) male patients. Meanwhile, there were 115 (87.1%) patients with positive HLA-B27. Regarding the biochemical indexes, the median [interquartile range (IQR)] C-reactive protein (CRP) and erythrocyte sedimentation rate (ESR) levels were 23.6 (16.4–38.7) mg/L and 28.0 (20.1–41.4) mm/h, respectively. As for disease activity scores, the median (IQR) BASDAI score was 5.3 (4.6–6.3), and the median ASDAS was 3.7 (2.9–4.2). Besides, the median (IQR) levels of TNF, IL-1β, and IL-6 were 75.5 (60.9–99.3) pg/ml, 3.1 (2.1–3.8) pg/ml, and 30.2 (24.4–39.4) pg/ml, respectively. Moreover, the median (IQR) proportion of Th1 and Th17 cells was 17.2 (14.5–22.2)% and 6.1 (4.8–7.6)%, respectively. Regarding treatment information, 120 (90.9%) patients had a history of NDAID treatment and 34 (25.8%) patients had a history of TNFi treatment; currently, 78 (59.1%) patients initiated NSAID treatment and 54 (40.9%) patients started TNFi treatment. More detailed clinical characteristics of patients with AS are shown in Table 1.

**Table 1:** Clinical characteristics of AS patients.

**Items**	**AS patients (*N* = 132)**
Age (years), mean ± SD	37.1 ± 9.7
Gender, No. (%)	
Female	14 (10.6)
Male	118 (89.4)
HLA-B27, No. (%)	
Negative	17 (12.9)
Positive	115 (87.1)
Biochemical indexes, median (IQR)	
CRP (mg/L)	23.6 (16.4–38.7)
ESR (mm/H)	28.0 (20.1–41.4)
Disease duration (years), median (IQR)	3.5 (0.2–6.2)
Disease activity scores, median (IQR)	
BASDAI score (0–10 cm VAS)	5.3 (4.6–6.3)
BASFI score (0–10 cm VAS)	4.5 (3.6–5.2)
Total back pain score (0–10 cm VAS)	6.0 (4.3–6.8)
PGADA score (0–10 cm VAS)	6.0 (5.0–7.0)
ASDAS	3.7 (2.9–4.2)
Proinflammatory cytokines, median (IQR)	
TNF (pg/mL)	75.5 (60.9–99.3)
IL-1β (pg/mL)	3.1 (2.1–3.8)
IL-6 (pg/mL)	30.2 (24.4–39.4)
Th cells and cytokines, median (IQR)	
Th1 cell (%)	17.2 (14.5–22.2)
IFN-γ (pg/mL)	9.0 (5.4–12.0)
Th17 cell (%)	6.1 (4.8–7.6)
IL-17A (pg/mL)	95.2 (68.9–124.0)
History of NSAID treatment, No. (%)	
No	12 (9.1)
Yes	120 (90.9)
History of TNFi treatment, No. (%)	
No	98 (74.2)
Yes	34 (25.8)
Current treatment, No. (%)	
NSAID treatment	78 (59.1)
TNFi treatment	54 (40.9)

*AS, ankylosing spondylitis; SD, standard deviation; IQR, interquartile range; HLA-B27, human leukocyte antigen B27; CRP, C-reactive protein; ESR, erythrocyte sedimentation rate; BASDAI, Bath Ankylosing Spondylitis Disease Activity Index; VAS, visual analog scale; BASFI, Bath Ankylosing Spondylitis Functional Index; PGADA, Patient Global Assessment of Disease Activity; ASDAS, Ankylosing Spondylitis Disease Activity Score; TNF, tumor necrosis factor; IL, interleukin; Th, T-helper; IFN, Interferon; NSAID, non-steroidal anti-inflammatory drug; TNFi, tumor necrosis factor inhibitor*.

### HDAC4 in Patients With AS, DCs, and HCs

**Figure 1:**
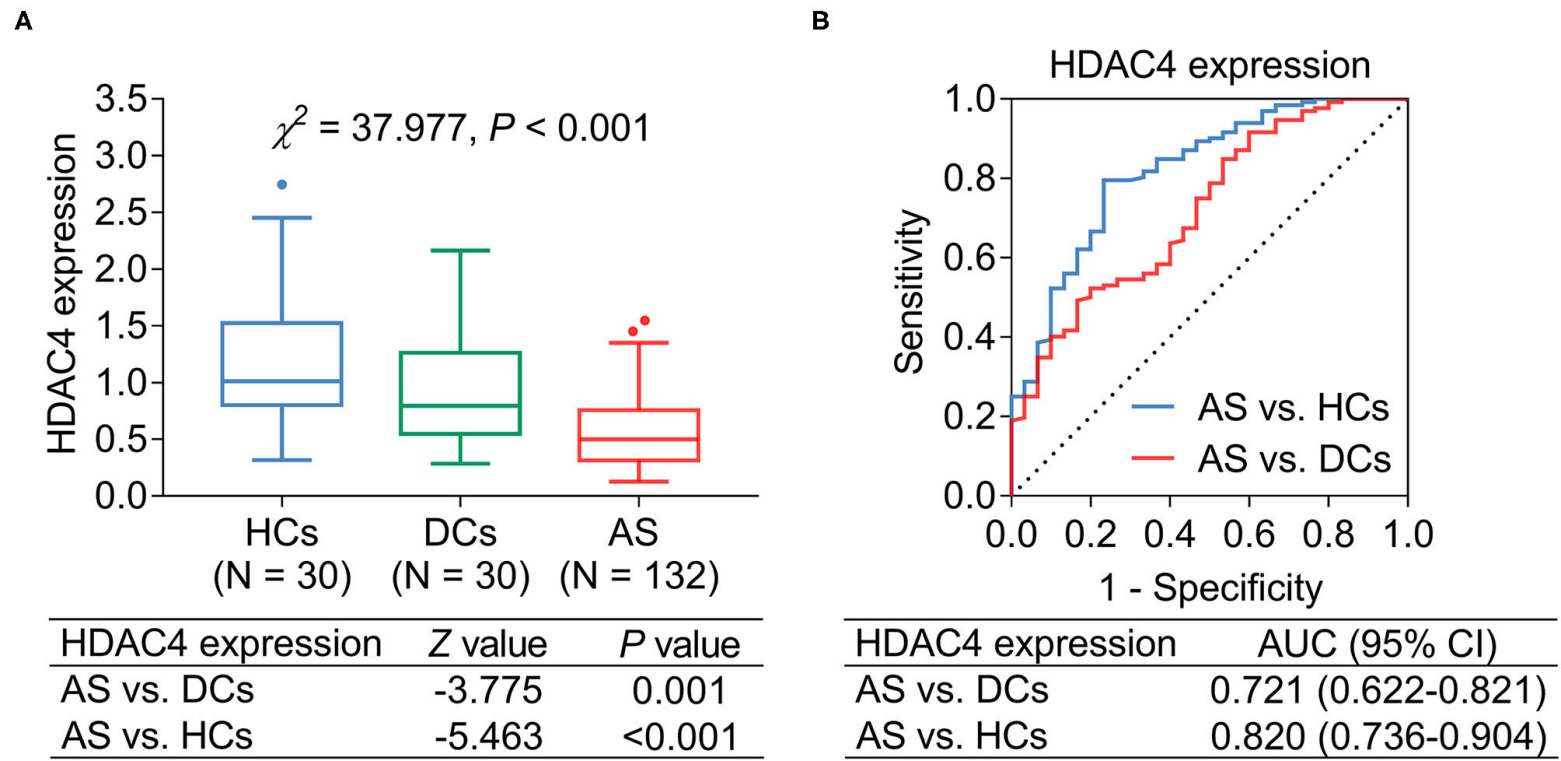
HDAC4 expression. HDAC4 expression in patients with AS, DCs, and HCs **(A)**; ROC curve analysis **(B)**.

Histone deacetylase 4 was decreased in patients with AS [median (IQR) level: 0.502 (0.313–0.758)] compared with DCs [median (IQR) level: 0.796 (0.546–1.262)] (*P* = 0.001) and HCs [median (IQR) level: 1.010 (0.800–1.524)] (*P* < 0.001) (Figure 1A). Subsequent ROC curve analysis showed that HDAC4 presented good ability in identifying AS patients from HCs [area under the curve (AUC): 0.820, 95% confidence interval (CI): 0.736–0.904]; meanwhile, HDAC4 also showed acceptable value in identifying patients with AS from DCs (AUC: 0.721, 95% CI: 0.622–0.821) (Figure 1B).

### Correlation of HDAC4 With Proinflammatory Cytokines, Th1 Cells, and Th17 Cells and Clinical Characteristics in Patients With AS

**Figure 2:**
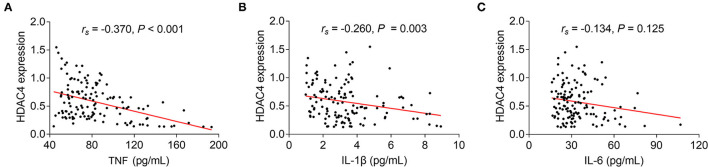
Correlation of HDAC4 with proinflammatory cytokines. Correlation of HDAC4 with TNF **(A)**, IL-1β **(B)**, and IL-6 **(C)** in patients with AS.

Regarding the correlation between HDAC4 and proinflammatory cytokines in AS patients, it was found that HDAC4 showed a strong, negative correlation with TNF (*r*_s_ = −0.370, *P* < 0.001) (Figure 2A) and a weak, negative correlation with IL-1β (*r*_s_ = −0.260, *P* = 0.003) (Figure 2B). However, HDAC4 did not correlate with IL-6 in patients with AS (*r*_s_ = 17 polarization 0.134, *P* = 0.125) (Figure 2C).

**Figure 3:**
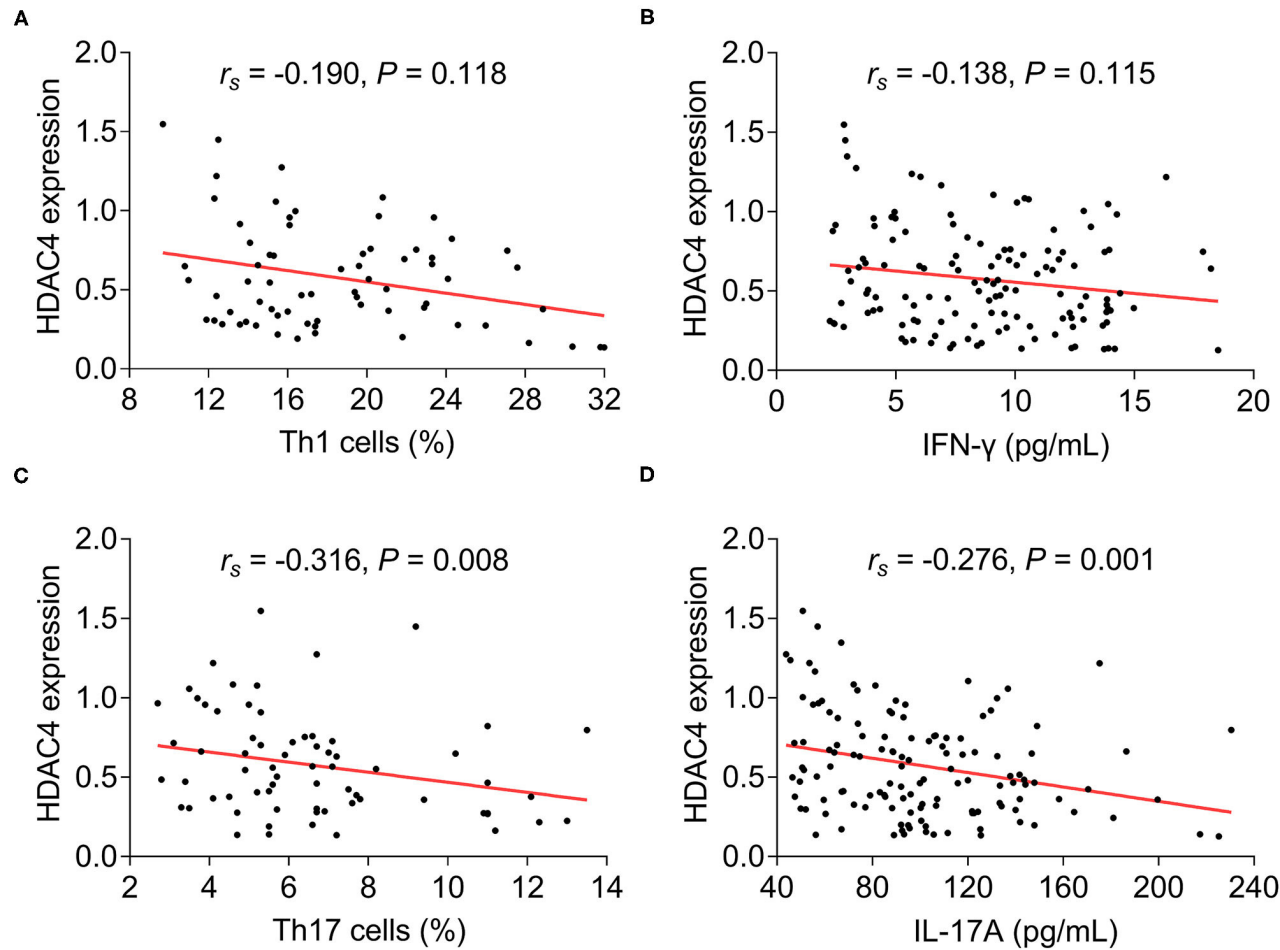
Correlation of HDAC4 with Th1 and Th17. Correlation of HDAC4 with Th1 cell proportion **(A)**, IFN-γ **(B)**, Th17 cell proportion **(C)**, and IL-17A **(D)** in patients with AS.

As to the correlation of HDAC4 with Th1 cells, Th17 cells, and their secreted cytokines in patients with AS, HDAC4 showed no correlation with Th1 cells (*r*_s_ = −0.190, *P* = 0.118) (Figure 3A) and their secreted cytokine IFN-γ (*r*_s_ = −0.138, *P* = 0.115) (Figure 3B), whereas HDAC4 was negatively correlated with Th17 cells (*r*_s_ = 0.316, *P* = 0.008) (Figure 3C) and their secreted cytokine IL-17A (*r*_s_ = −0.276, *P* = 0.001) (Figure 3D) in patients with AS.

In terms of the association of HDAC4 with categorized characteristics of AS patients, no association was found in HDAC4 with gender, HLA-B27, and history of NSAID or TNFi (all *P* > 0.05) (Table 2). Concerning the correlation of HDAC4 with continuous characteristics of patients with AS, HDAC4 was negatively correlated with CRP (*r*_s_ = −0.349, *P* < 0.001) and ASDAS (*r*_s_ = −0.181, *P* = 0.038), but not with other characteristics including ESR, disease duration, BASDAI score, BASFI score, total back pain score, or patient global assessment of disease activity score (all *P* > 0.05) (Table 2).

**Table 2:** Correlation of HDAC4 expression with disease characteristics among AS patients.

**Items**	**HDAC4 expression**	***Z* or *r_***s***_* value**	***P*-value**
	**Median (IQR)**		
Gender		−0.336	0.737
Female	0.425 (0.291–0.774)		
Male	0.506 (0.316–0.759)		
HLA-B27		−0.082	0.935
Negative	0.546 (0.324–0.719)		
Positive	0.486 (0.307–0.763)		
History of NSAID		−1.175	0.240
No	0.401 (0.145–0.743)		
Yes	0.506 (0.328–0.758)		
History of TNFi		−1.918	0.055
No	0.485 (0.306–0.717)		
Yes	0.590 (0.323–1.015)		
Age	–	−0.056	0.526
CRP	–	−0.349	<0.001
ESR	–	−0.110	0.210
Disease duration	–	−0.073	0.406
BASDAI score	–	−0.155	0.076
BASFI score	–	−0.162	0.063
Total back pain score	–	−0.146	0.095
PGADA score	–	−0.132	0.132
ASDAS	–	−0.181	0.038

*HDAC4, Histone deacetylase 4; AS, ankylosing spondylitis; IQR, interquartile range; HLA-B27, human leukocyte antigen-B27; NSAID, nonsteroidal anti-inflammatory drug; TNFi, tumor necrosis factor inhibitor; CRP, C-reactive protein; ESR, erythrocyte sedimentation rate; BASDAI, Bath Ankylosing Spondylitis Disease Activity Index; BASFI, Bath Ankylosing Spondylitis Functional Index; PGADA, Patient Global Assessment of Disease Activity; ASDAS, Ankylosing Spondylitis Disease Activity Score*.

### Longitudinal Change of HDAC4 in Patients With AS

**Figure 4:**
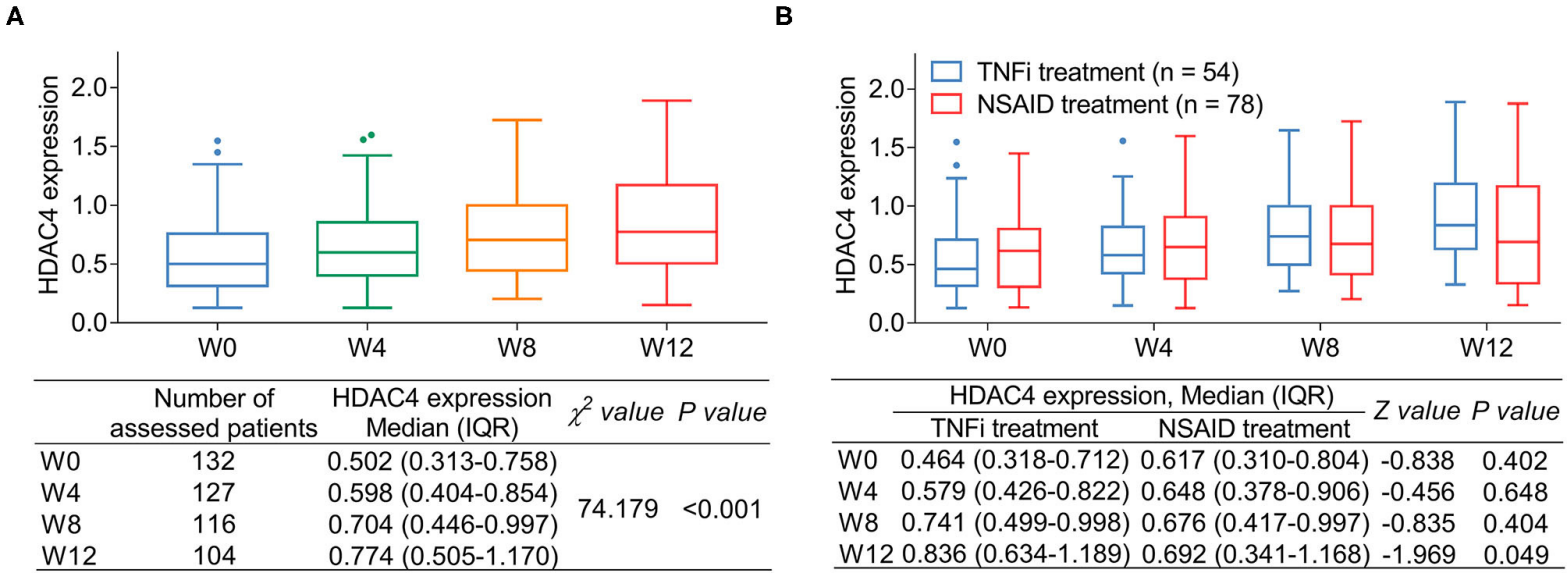
Change in HDAC4 after treatment. Change in HDAC4 from W0 to W12 after treatment in patients with AS **(A)**. *P*-value representing the difference of HDAC4 among different time points (W0, W4, W8, and W12). Comparison of HDAC4 in patients with AS receiving TNFi or NDAID **(B)**. *P*-value representing the difference of HDAC4 at W0, W4, W8, and W12 between TNFi group and NSAID group.

Histone deacetylase 4 was different among W0, W4, W8, and W12, which presented an increasing trend from W0 to W12 in patients with (*P* < 0.001) (Figure 4A). Meanwhile, comparison analysis was conducted between patients with AS receiving TNFi or NSAID treatment, which presented that HDAC4 at W12 was increased in patients with AS receiving TNFi treatment compared with those receiving NSAID treatment (*P* = 0.049); however, HDAC4 at W0, W4, and W8 remained similar between these two types of patients (all *P* > 0.05) (Figure 4B).

### Association of HDAC4 With Treatment Response in Patients With AS

**Figure 5:**
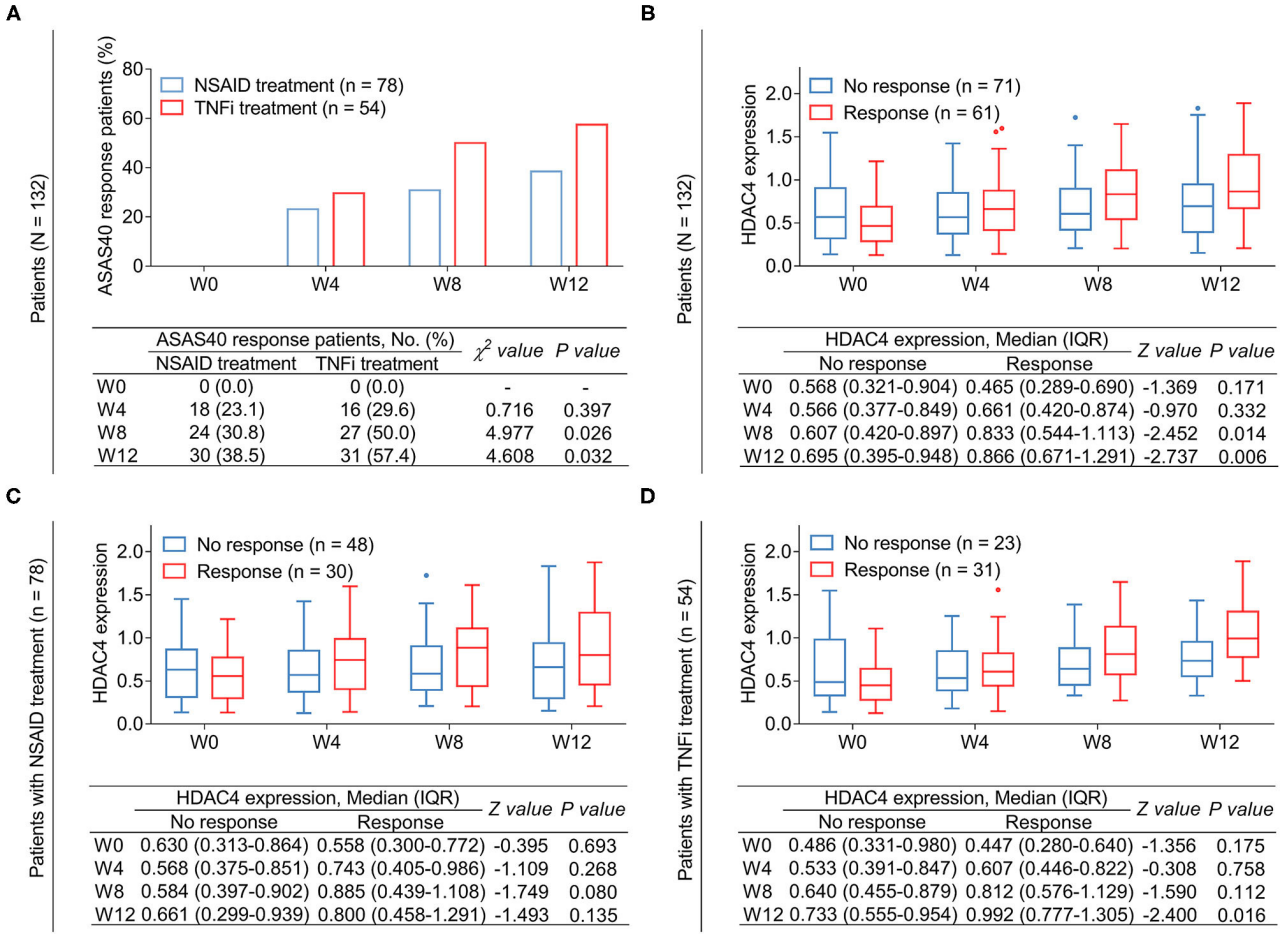
Correlation of HDAC4 with treatment response. Comparison of response rate at different time points between patients with AS receiving TNFi and those receiving NSAID **(A)**. *P*-value representing the difference of the proportion of ASAS40 response patients at W0, W4, W8, and W12 between TNFi group and NSAID group. Comparison of HDAC4 between responder at W12 and non-responder at W12 in total AS patients **(B)**. Comparison of HDAC4 between responder at W12 and non-responder at W12 in patients with AS who received TNFi **(C)**. Comparison of HDAC4 between responder at W12 and non-responder at W12 in patients with AS who received NSAID **(D)**. *P*-value in **(B–D)** representing the difference of HDAC4 at W0, W4, W8, and W12 between responders and non-responders.

The number of patients who achieved ASAS40 response at W0, W4, W8, and W12 were 0 (0.0%), 16 (29.6%), 27 (50.0%), and 31 (57.4%), respectively, in TNFi-treated patients and 0 (0.0%), 18 (23.1%), 24 (30.8%), and 30 (38.5%) in NSAID-treated patients. Further comparison analysis revealed that the proportion of patients who achieved ASAS40 response was higher at W8 (*P* = 0.026) and W12 (*P* = 0.032) but remained similar at W4 (*P* = 0.397) in patients receiving TNFi treatment compared with those receiving NSAID treatment (Figure 5A).

Subsequently, the relationship between HDAC4 and treatment response in patients with AS was analyzed. Data revealed that among all patients with AS, HDAC4 at W8 (*P* = 0.014) and W12 (*P* = 0.006) was higher in response patients compared with non-response patients (Figure 5B). By subgroup analysis, after NSAID treatment, HDAC4 at all time points remained similar between response and non-response patients (all *P* > 0.05) (Figure 5C); meanwhile, after TNFi treatment, HDAC4 at W12 was elevated in response patients compared with non-response patients (*P* = 0.016) (Figure 5D).

### Effect of HDAC4 on Th Cell Polarization in AS

**Figure 6:**
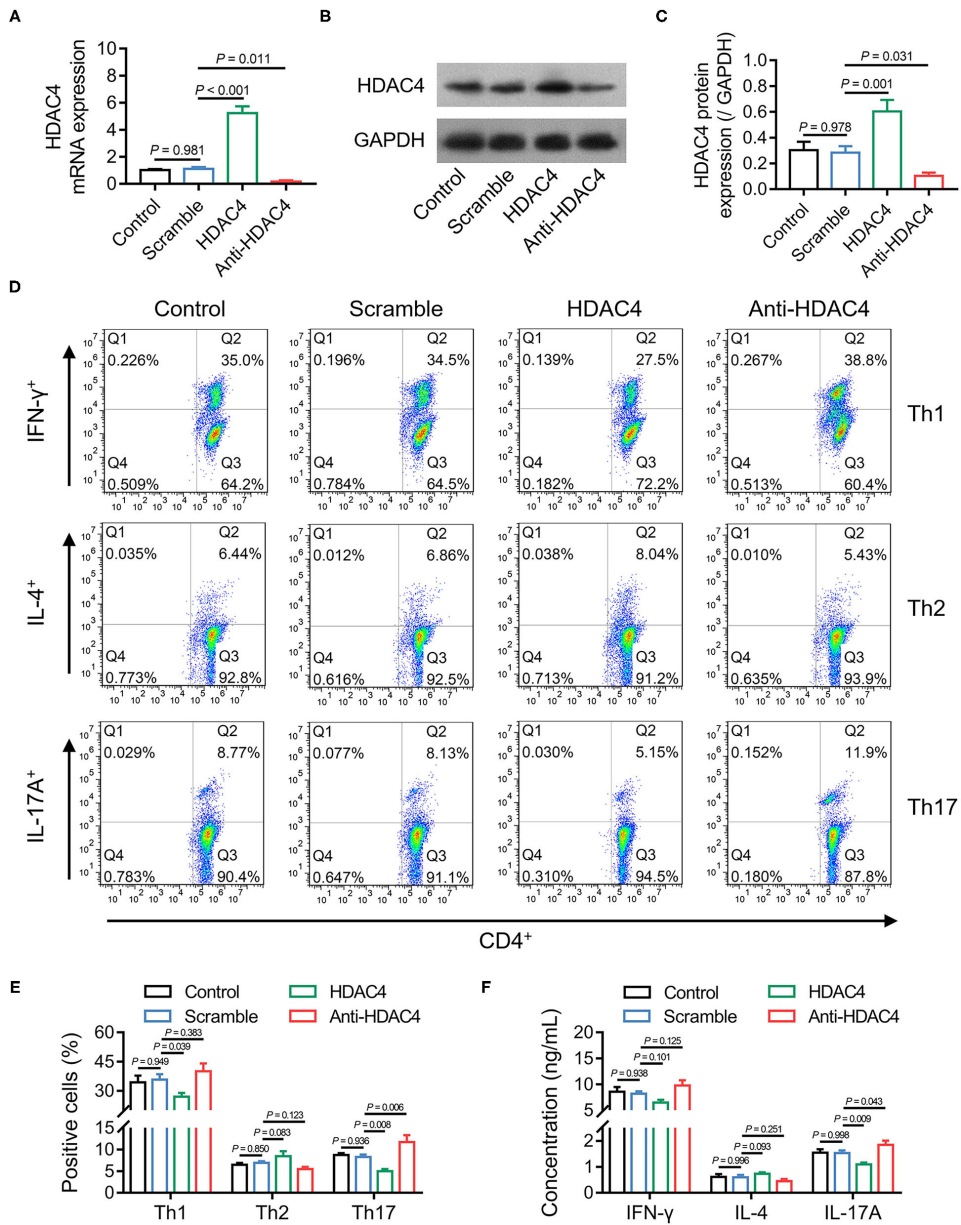
HDAC4 negatively regulated Th17 polarization. Comparison of HDAC4 mRNA expression after transfection **(A)**. Detection of HDAC4 protein expression after transfection **(B)**. Comparison of HDAC4 protein expression after transfection **(C)**. Detection of Th1, Th2, and Th17 polarization after transfection **(D)**. Comparison of Th1, Th2, and Th17 cell proportion after transfection **(E)**. Comparison of IFN-γ, IL-4, and IL-17A after transfection **(F)**.

Th cell polarization is one of the critical regulators of AS; meanwhile, the data mentioned earlier presented that HDAC4 was correlated with Th17 cells in patients with AS. Therefore, whether HDAC4 could affect Th cell polarization in AS was further explored. Initially, naïve CD4^+^ T cells from patients with AS were isolated and transfected with HDAC4 overexpression or knockdown lentivirus. Then, RT-qPCR and Western blot analysis confirmed high transfection efficiency (Figures 6A–C). Subsequently, the transfected naïve CD4^+^ T cells were, respectively, polarized into Th1, Th2, and Th17 cells. The flow cytometry analysis revealed that HDAC4 overexpression reduced Th1 (*P* = 0.039) and Th17 (*P* = 0.008) cell percentage, but not Th2 cell percentage (*P* = 0.083); whereas HDAC4 knockdown only promoted Th17 cell percentage (*P* = 0.006) (Figures 6D,E). Meanwhile, the ELISA analysis revealed that HDAC4 overexpression decreased, while its knockdown increased IL-17A level (both *P* < 0.05); however, they could not affect IFN-γ or IL-4 level (all *P* > 0.05) (Figure 6F). These data suggested that HDAC4 negatively regulated Th17 polarization but has a weak effect on Th1 or Th2 polarization in AS.

## Discussion

Histone deacetylase 4 is a critical regulator of various biological processes, including inflammation, T cell differentiation, and malignant behavior of cancer cells; thus, previous studies have reported the role of HDAC4 as a biomarker in multiple diseases (13, 17, 23). For instance, it has been suggested that HDAC4 is negatively correlated with prognosis in patients with cancers, including ovarian cancer, esophageal carcinoma, and hepatocellular carcinoma (24–26). Meanwhile, hypermethylation of HDAC4 is associated with severe liver fibrosis in patients with hepatitis B-related chronic liver disease (27). Regarding the abnormal expression of HDAC4 in patients with autoimmune diseases, it has been revealed that HDAC4 is decreased in the synovial tissues from rheumatoid arthritis patients compared with those from healthy subjects (12); in addition, HDAC4 is also reduced in the CD4^+^ T cells and intestinal mucosa tissues in patients with inflammatory bowel disease compared with those from healthy controls (16). However, whether HDAC4 also presents abnormal expression in patients with AS is unclear. This study revealed that HDAC4 was reduced in patients with AS compared with HCs and DCs; meanwhile, HDAC4 could distinguish patients with AS from HCs and DCs. Possible explanations could be that: (1) low expression of HDAC4 might promote the differentiation of Th17 cells and inflammation, thus leading to AS (2, 18, 28) in this study, patients with OA were served as DCs. Compared with patients with OA, patients with AS are characterized by immune system dysregulation (29, 30). Meanwhile, because HDAC4 can regulate the development and functions of immune cells (16, 31), it might reflect the degree of immune disorder to some extent. Therefore, HDAC4 could distinguish patients with AS from DCs.

Ankylosing spondylitis is characterized by the clinical symptoms of back pain, immobility in the spinal area, and a high degree of inflammation, which mainly affects the quality of life of patients with AS (1). Several biomarkers have been identified to promptly monitor inflammation and disease activity, thus improving the management of patients with AS. For example, it has been revealed that dual-specificity protein phosphatase 22 is negatively correlated with proinflammatory cytokines and disease activity in patients with AS (32). Meanwhile, long non-coding RNA intersectin 1–2 from the PBMCs of patients with AS shows a relatively weak correlation with ASDAS (33). However, the correlation of HDAC4 with disease characteristics of patients with AS is not clear. Our data proposed that HDAC4 was negatively correlated with inflammation level and disease activity in patients with AS. That could interpret these data: (1) according to previous studies, HDAC4 inhibits inflammation in rheumatoid arthritis and inflammatory bowel disease (12, 17), suggesting that HDAC4 might be a critical negative regulator of inflammation in autoimmune diseases; therefore, HDAC4 was negatively associated with inflammation in patients with AS in our study; (2) low level of HDAC4 might exacerbate inflammation to aggravate the damage in the joints, thus leading to increased pain in patients with AS, while inflammation (reflected by CRP or ESR), back pain, and peripheral pain/swelling are assessment criteria of ASDAS. Therefore, HDAC4 was negatively correlated with ASDAS in patients with AS.

Th17 cells have been reported to be critically involved in the pathogenesis of AS (34). Apart from the preclinical investigation, a recent meta-analysis involving 95 articles found that Th17 cells were elevated in patients with AS compared with controls (35). Moreover, it has also been suggested that reduction of Th17 cells is associated with response to TNFi in patients with AS (36). In this study, HDAC4 was found to be negatively associated with Th17 cell proportion in patients with AS. Furthermore, *in vitro* investigations presented that HDAC4 negatively regulated the polarization of Th17 cells in naïve CD4^+^ T cells of patients with AS, which was in line with a previous study focusing on inflammatory bowel disease (16). We deduced that HDAC4 might regulate some pathways, such as the c-Jun N-terminal kinase pathway, to regulate the production of several cytokines, including IL-1β, IL-6, IL-23, etc., which were critical for the polarization of CD4^+^ T cells into Th17 (37, 38). However, this should be further verified. Also, these data could explain the negative correlation between HDAC4 and Th17 cell proportion in patients with AS. However, our data also suggested that HDAC4 could not affect the polarization of Th1 or Th2 from naïve CD4^+^ T cells of patients with AS, while further investigation is needed.

According to previous studies, there exists a certain proportion of patients with AS who may not respond to the treatments (8). One of the solutions to improve this circumstance is to search for potential biomarkers of treatment response, thus improving the management of AS. In our study, HDAC4 was found to be progressively elevated after treatment. A possible explanation for this might be that: according to the data as mentioned above, HDAC4 was negatively correlated with disease activity in patients with AS; meanwhile, disease activity gradually decreased in patients with AS after treatment, and therefore, HDAC4 was increased after treatment. Meanwhile, the elevation of HDAC4 was associated with treatment response in total AS patients. To further explore this issue, subgroup analysis was conducted, which presented that elevated HDAC4 was associated with treatment response in TNFi-treated patients but not in NSAID-treated patients. The possible explanation could be that TNFi possessed a more substantial inhibitory effect on inflammation than NSAID, which may lead to a rapid elevation in HDAC4, whereas NSAID had a weaker effect. Therefore, the increase in HDAC4 was associated with treatment response in TNFi-treated patients. In this study, patients in the NSAID group receive meloxicam 7.5 mg/day, celecoxib 200 mg/day, or imrecoxib 100 mg two times daily, which is a relatively low dose. Although in patients with AS, the dose of meloxicam 7.5 mg or 15 mg/day and the dose of celecoxib 200 mg or 400 mg/day are all reported (39, 40), a relatively low dosage of NSAID might affect the treatment response in NSAID group.

There existed several limitations of this study. First, our data presented that HDAC4 was negatively correlated with Th17 cells and IL-17A, inspiring that the relationship between HDAC4 and treatment response to IL-17A inhibitor could be further investigated. Second, the molecular mechanism of HDAC4 implicated in the pathogenesis of AS could be investigated in further studies. Third, the relationship between HDAC4 and the disease progression and treatment response in other autoimmune diseases could be further explored.

Collectively, HDAC4 is negatively correlated with inflammation disease activity, and it reversely modulates Th17 polarization, whose elevation after treatment links to treatment response in patients with AS.

## Data Availability Statement

The original contributions presented in the study are included in the article/supplementary material, further inquiries can be directed to the corresponding author/s.

## Ethics Statement

The studies involving human participants were reviewed and approved by The First Hospital of Jilin University. The patients/participants provided their written informed consent to participate in this study.

## Author Contributions

ZJ and LZ conceived and designed the study and edited the manuscript. BD, ZJ, and LZ collected and analyzed the data. BD and FM prepared the figures tables and wrote the manuscript. All authors revised the manuscript and read and approved the submitted version.

## Funding

We thank the staff of the Division of Clinical Research and the Genetic Diagnosis Center in our hospital for their assistance during this study. This work was supported by Jilin Province Direct Health Special Project (JLSWSRCZX2021-062); Jilin Province Direct Health Special Project (JLSWSRCZX2020-038); the National Natural Science Foundation of China (Grant Number 81501343); the Bethune Plan Project of Jilin University (Grant Number 2015410); the Jilin Scientific and Technological Development Program (Grant Number 20170520010JH; 20150101152JC); the Postdoctoral Science Foundation of China (Grant Number 801181010432).

## Conflict of Interest

The authors declare that the research was conducted in the absence of any commercial or financial relationships that could be construed as a potential conflict of interest.

## Publisher's Note

All claims expressed in this article are solely those of the authors and do not necessarily represent those of their affiliated organizations, or those of the publisher, the editors and the reviewers. Any product that may be evaluated in this article, or claim that may be made by its manufacturer, is not guaranteed or endorsed by the publisher.
